# Scarce resources, public health and professional care: the COVID-19 pandemic exacerbating bioethical conflicts — findings from global qualitative expert interviews

**DOI:** 10.1186/s12889-023-17249-4

**Published:** 2023-12-13

**Authors:** Jane Vonderschmitt, Sabine Wöhlke, Silke Schicktanz

**Affiliations:** 1 Department of Medical Ethics and History of Medicine, University Medical Center Goettingen, Humboldtallee 36 / 37073 Goettingen, Germany; 2 Department of Health Sciences, Hamburg University of Applied Sciences, Ulmenliet 20 / 21033 Hamburg, Germany

**Keywords:** COVID-19, Pandemic, Bioethics, Vulnerability, Global Comparison, Experts, Qualitative Interviews

## Abstract

**Background:**

Since spring 2020, the SARS-CoV-2 virus has spread worldwide, causing dramatic global consequences in terms of medical, care, economic, cultural and bioethical dimensions. Although the resulting conflicts initially appeared to be quite similar in most countries, a closer look reveals a country-specific intensification and differentiation of issues. Our study focused on understanding and highlighting bioethical conflicts that were triggered, exposed or intensified by the COVID-19 pandemic in low and middle-income countries (LMICs) and high-income countries (HICs).

**Methods:**

We conducted qualitative interviews with 39 ethics experts from 34 countries (Argentina, Australia, Austria, Brazil, Canada, Colombia, Denmark, Ecuador, Ethiopia, France, Germany, India, Italy, Israel, Japan, Kyrgyzstan, Mexico, Nigeria, Oman, Pakistan, Paraguay, Poland, Romania, Russia, Singapore, South Korea, Spain, Sweden, South Africa, Tunisia, Türkiye, United-Kingdom, United States of America, Zambia) from November 2020 to March 2021. We analysed the interviews using qualitative content analysis.

**Results:**

The scale of the bioethical challenges between countries differed, as did coping strategies for meeting these challenges. Data analysis focused on:

- Resource scarcity in clinical contexts: Scarcity of medical resources led to the need to prioritize the care of some COVID-19 patients in clinical settings globally. Because this entails the postponement of treatment for other patients, the possibility of serious present or future harm to deprioritized patients was identified as a relevant issue.
- Health literacy: The pandemic demonstrated the significance of health literacy and its influence on the effective implementation of health measures.
- Inequality and vulnerable groups: The pandemic highlighted the context-sensitivity and intersectionality of the vulnerabilities of women and children in LMICs and the aged in HICs.
- Conflicts surrounding healthcare professionals: The COVID-19 outbreak underscored the tough working conditions for nurses and other health professionals, raising awareness of the need for reform.

**Conclusion:**

The pandemic exposed pre-existing structural problems in LMICs and HICs. Without neglecting individual contextual factors in the observed countries, we created a mosaic of different voices of experts in bioethics across the globe, drawing attention to the need for international solidarity in the context of a global crisis.

**Supplementary Information:**

The online version contains supplementary material available at 10.1186/s12889-023-17249-4.

## Introduction

Severe acute respiratory syndrome coronavirus 2 (SARS-CoV-2), the virus causing COVID-19, was first detected in December 2019 in Wuhan, China. Its rapid spread to many other countries led the World Health Organization (WHO) to declare the international developments surrounding the virus to be a pandemic on March 11, 2020 [1]. Worldwide, the COVID-19 outbreak had serious health consequences including long hospitalizations and high mortality rates [2, 3]. Many countries responded by implementing public health (PH) strategies to control COVID-19 outbreaks, using policies that included identifying and quarantining infected individuals, mandating the wearing of protective masks, social distancing and restrictions on international travel [4, 5]. PH strategies gave rise to a multitude of bioethical conflicts and challenges across the globe, and this study investigated their similarities and differences drawing on expert interviews. “Bioethics” is used in the study as an umbrella term for ethical issues, problems and dilemmas arising in the fields of biology, research, medicine, PH and healthcare [6]. Depending on the context, we differentiate between medical ethics (all clinical settings), PH ethics (collective issues) and nursing ethics specifically. To answer our research question of how, in cross-national comparison, bioethical conflicts were triggered or exacerbated by the COVID-19 pandemic, we categorized our findings into four main topics including A) resource scarcity in medical contexts, B) health literacy, C) inequality and vulnerable groups and D) conflicts surrounding health-care professionals [5, 7]. These ethical categories were established deductively from empirical findings; their theoretical foundations had been outlined previously, between June and November 2020. We distinguished between low and middle-income countries (LMICs) and high-income countries (HICs) by using the World Bank classification of countries based on Gross National Income: lower-income (< $1,085), lower-middle income ($1,085—$4,256), upper-middle income ($4,256—$13,205) and high-income (> $13,205) [8].

## Background

COVID-19 created or exposed various conflicts of resource scarcity in clinical settings as decisions about the allocation of scarce resources had to be made. While this situation has been widely discussed in HICs especially as ‘triage’ in the clinical setting [9–12], in other countries, particularly in LMICs, prioritization decisions entailed also excluding individuals from basic healthcare access completely [13]. Under conditions of resource scarcity in clinical settings, some countries formalized priorities in the allocation of life-saving resources, such as ventilators and ICU beds, favouring certain populations [11, 14]. In this context, the prioritization of COVID-19-related cases also led to the postponement of other treatments [15], for example tuberculosis (TB) and human immunodeficiency virus (HIV) in Congo [16]. This raises potential bioethical conflicts of resource allocation.

PH ethical dilemmas principally derive from the tensions between the public good and restrictions on personal freedom [17]. Infection-control measures like masks and contact restrictions, for example, were imposed to protect population health but were often criticized as an unjustifiable curtailment of individual freedom. While the intensity of contact tracing in countries such as Israel and South Korea led to discussions about data protection and protests against interventions intended to reduce virus spread [18, 19], debates about vaccines ranged from possible vaccination mandates [20] to global equitable distribution and national prioritization [14, 21]. Health literacy – the skills to understand and to use relevant health information – has been identified as an essential determinant of public acceptance of measures [22]. Yet, the promotion of health literacy has become increasingly complicated in the twenty-first century due to the spread of disinformation and conspiracy theories in social media, leading to a decrease in the acceptance of important PH interventions [22, 23].

Vulnerability is understood as a state of being at increased risk of harm with reduced capacity to protect oneself [24] and is also a component of ethical debate. In the context of COVID-19, social and medical inequalities in the discriminatory attitudes towards older people in HICs have become apparent, giving rise to further ethical issues [25, 26]. Meanwhile, in Latin American countries the protection of women and children was discussed during the first wave of the pandemic [27]. Other literature identifies conflicts around additional vulnerable groups, such as people with disabilities and ethnical minorities, as a pressing global issue [28].

Finally, health-care professionals^1^ gloablly experienced specific ‘frontline’ stressors [29], including, for example, high workloads under scarcity of staff, time and protective equipment [12, 30–32]. These added tensions strained the integrity of professional standards, particularly in nursing, that are already at risk due to permanently difficult working conditions [31]. They created sometimes contradictory political demands and high societal expectations [12]. Additionally, various challenging situations, such as patient mortality and the safety of colleagues and families, further increased mental stress. Moral dilemmas were more frequent, resulting in ‘moral distress’ [33] for many health professionals.

Through our study of expert opinion, we provide an exploratory contribution that takes a global perspective. It complements existing studies, which focus predominantly on single countries. We conducted interviews with 39 experts in bioethics (including clinical ethics) or PH ethics. A third of these experts are addtionally involved in medical research or policy-making related to COVID-19. Their expertise in relevant topics provides valuable insights into the realities of different countries and cannot be adequately captured quantitatively. By comparing their appraisals of the bioethical issues of COVID-19, we hope to open a globalised perspective on ethical issues for future pandemics.

## Methods and materials

### Study design

For this exploratory study we used qualitative research methods to identify bioethical conflicts created or revealed by the COVID-19 pandemic. We conducted 39 semi-structured expert interviews. The aim of our qualitative study was to fill the gap of a global perspective by classifying commonalities and differences perceived in bioethical conflicts in context of the COVID-19-pandemic in 34 countries. The study is part of a larger project at the Department of Medical Ethics of the University of Göttingen that has presented the experts’ assessments in an internet-based virtual exhibition since May 2022. It is publicly accessable in the form of podcasts and interactive teaching material.^2^ The study was approved by the Ethics Committee of the University Medical Center Göttingen (32/10/20).

### Sampling, data collection, consent

Recruitment of experts took place from November 2020 through January 2021 and was based on the department’s exiting international network of scientists, a targeted internet search and an emergent snowball system using recommendations from third parties and interviewed experts. Participants were selected regarding their involvement in public discourses concerning COVID-19, such as published papers and participation in international online sessions on COVID-19, their spectrum of research and country of residence. Some were representatives on ethics committees, some were lecturers on the pandemic and others were involved in clinical practice as physicians and spoke of their ‘front-line’ experience. To ensure confidentiality, we give no details regarding the professional status of interviewees, but some have more clinical expertise and others a more theoretical background.

In total, we conducted 39 interviews with experts of different ethical fields, reporting from 34 different countries. The differentiation of the countries studied into LMICs and HICs was based on the World Bank’s classification.^3^ Table 1 provides an overview of the experts interviewed.

**Table 1:** Participant characteristics (*n* = 39)

Characteristics	% (n)
**Gender**	
Male	53.8 (21)
Female	46.2 (18)
**Field of ethical expertise**	
Bioethics (including clinical ethics)	78.0 (29)
Public health ethics	22.0 (11)
Parallel working: Research ethics (6), physicians (8), politics (5)	31.6 (19)
**Experts stemming from the following continents**	
**Europe** Austria, Denmark, Germany, France, Italy, Spain, Sweden, Poland, Romania, UK	28.2 (11)
**Asia** India, Israel, Japan, Kyrgyzstan, Oman, Pakistan, Russia, Singapore, South Korea, Türkiye	25.6 (10)
**Africa** Congo, Ethiopia, Nigeria, South Africa, Tunisia, Zambia	15.4 (6)
**America** Argentina, Brazil, Canada, Colombia, Ecuador, Mexico, Paraguay, USA	25.6 (10)
**Oceania** Australia	5.2 (2)

We collected the data in form of semi-structured expert interviews. With reference to a literature review of publications up to November 2020 regarding potential ethical conflicts worldwide in context of COVID-19, a standardized semi-structured interview guide was formulated, aiming to structure the content by means of 13 open-ended questions regarding scarce resources, PH and professional care (including further sub-topics, see Additional file 1: Interview guideline). The guide was pretested in three interviews to ensure its validity and comprehensibility and sent to participants prior to the interviews.

Data collection took place online via Zoom^4^ between November 2020 and March 2021. All interviews were conducted by J.V. with the majority in English (35), but also in German (3) and Spanish (1). The average interview lasted about 50 min. Prior to data collection, written and verbal informed consent for data recording and storage was obtained from all participants. For the next analytical step, recordings and transcripts were then pseudonymized and anonymized for publication.

### Data analysis

Data was analysed by means of qualitative content analysis [34]. The material was reproduced verbatim and, if necessary, translated into English. Analysis of the material involved the language-condensation and coding of the transcripts. The coding guideline (see Additional file 2: Code overview), had been created deductively following a literature review. Additionally, inductive codes were added during the coding process and further compared with existing research. The coding guideline was pretested by peer-coding before application. Furthermore, 25% of the collected material was peer-coded by the co-authors and one external colleague.

The coding process was performed using the software Atlas.ti™.^5^ In a first run of the material review, 16 so-called *main codes* were created (see Additional file 2: Code overview), which already represented general terms for selected quotations or context units taken from the text. In several condensing steps, irrelevant information and repetitions were deleted and a transformation of the text on a uniform language level took place. Condensing steps were always carried out with consideration of the theoretical background. For more detailed access to the text, these main codes were subsequently expanded, specified and revised, always in light of the research question. Hence, in addition to the main codes, 43 sub-codes (see Additional file 2: Code overview) were inductively created and added to the coding guideline. Subsequently, a reduction and abstraction of the coded text passages was performed to finalize the category system. Coded text passages were then thematically interpreted, supported with anchor examples and summarized in text form, always identifying the countries.

## Results

Our data analysis identified a wide range of bioethical conflicts. For analysis, we structured this range using three main topics: scarce resources, public health and professional care*.* Each main theme includes more than one subtheme as summarized in Table 2. For transparency and contextual comprehensibility, we mention the countries related to the experts’ statements. For simplification, we grouped the countries into HICs and LMICs, as national income impacts national healthcare systems. These findings are described below (see also Additional file 3: Results summary):

**Table 2:** Overview themes and subthemes

**A) Effects of scarcity for the clinical context**
A1) Different dimensions of resource scarcity between LMICs and HICs
A2) Prioritization of COVID-19 patients
**B) Health literacy**
**C) Inequality and vulnerable groups**
C1) Poverty as a specific challenge for LMICs
C2) Children and women as a specific vulnerable group in LMICs
C3) Older people as a specific vulnerable group in HICS
**D) Conflicts surrounding health-care professionals**
D1) Frontline burdens
D2) Psychological and moral distress

### Effects of scarcity for the clinical context

Concerning the scarcity of resources and the consequences of prioritization in the medical-clinical context during the COVID-19 pandemic, ethical problems of global, national and regional scope became apparent. We present them in the following.

#### Different dimensions of resource scarcity between LMICs and HICs

According to all interviewed experts, there was a pronounced lack of material resources in the clinical setting, especially at the beginning of the pandemic. This included COVID-19 tests and personal protective equipment (PPE). In the context of international discrepancies between LMICs and HICs, experts from Ecuador, Ethiopia and Türkiye emphasized the unequal conditions in terms of access to the international market.

From a national perspective, regardless of the interviewed experts’ origin, the majority identified the lack of PPE in clinics and nursing homes as one of the biggest problems. Experts from Russia, Oman, Zambia and Argentina, among others, mentioned the resulting serious COVID-19 infections and COVID-related deaths among healthcare personnel themselves. Furthermore, interview partners from the African LMICs Zambia, Ethiopia and Tunisia reported that the severe lack of PPE resulted in extreme re-usage of used materials which can be problematic for hygienic reasons.

*‘PPE has been a huge issue here. [...] I didn’t have any disposable PPE, so I had to use my regular one [...]. I had people with COVID, so I put it [PPE] in plastic, took it home and washed it and I will use it again’. (Zambia)*

Emphasizing that long-existing financial deficiencies in healthcare systems became more apparent, experts from the LMICs Ethiopia, Paraguay, Ecuador, Pakistan and Kyrgyzstan reported serious scarcities in terms of basic resources. Additionally, other experts from LMICs like Mexico, Kyrgyzstan, Paraguay, Ecuador and South Africa emphasized a fundamental lack of health infrastructure and ICU equipment in local hospitals that had already existed before the crisis:

*‘From the beginning it was clear that the public clinics wouldn't cope, that there were no near enough ICU beds in the country based on the projections that we had at that time. I think South Africa has about 3000 ICU beds in total and most of those are in the private healthcare system which most people can’t access’. (South Africa)*

Interviewees from Pakistan, Kyrgyzstan, Paraguay, Romania and Japan accentuated deficits of resources especially in rural health facilities. Compared to urban clinics, small provincial ones were not as well supplied during the pandemic, which resulted in a national and inter-clinical inequality of providing medical care.

#### Prioritization of COVID-19 patients

The severe shortage of material but also of personnel resources in clinics resulted in the prioritization of COVID-19 patients over other patients who, consequently, were harmed. The majority of experts reported a focus of medical care on COVID-19 and emergency medicine, especially during the initial period. Though shortage of medical material was mentioned by every interview partner, reported resource allocation and consequences of priority-setting varied across the countries. Especially in HICs, such as Austria, the United Kingdom and Canada, the main strategy was to postpone surgery interventions and non-essential treatments. Meanwhile, in India, Brazil, Argentina and Italy entire hospitals were transformed into exclusive COVID-19 facilities or even totally closed to redistribute available resources:

*‘Many hospitals have been completely closed because of the pandemic and the patient, who usually went to this hospital, hadn't the possibility to be treated as usual. This was a big problem’. (Italy)*

The transformation of hospitals into COVID-19 facilities led to conflicts worldwide regarding equal access to treatment options during the pandemic. An expert from Sweden concluded that *‘prioritizing one group means making another one suffer’.*

Most experts considered two sides. On the one hand, there has been a prioritization of emergencies and COVID-19 cases by policy and/or by the healthcare facilities themselves, aiming to minimize infections and to prevent a collapse of the entire system. On the other hand, patients themselves refused to visit potential infectious institutions, such as clinics. Interview partners from HICs such as Austria, France and Sweden, also reported a general mobilization of resources towards COVID-19, which resulted in an unequal distribution of available resources on different wards in hospitals. By limiting elective procedures, screenings and the overall number of patients in general, an attempt was made to prevent the system from becoming overburdened. Many experts reported that this *‘Covidization’* (EC17) of healthcare systems resulted in a global treatment deficit, especially for oncological patients all over the world. Delayed diagnosis of malignant tumours due to reduced screening, diagnosis and control examinations, potentially has led to an increase in undetected tumours, as an expert from Oman with clinical experience stated:

*‘We had [oncological] patients who were delayed three months because their previous appointment was cancelled because it was thought to be an elective procedure, [but] it turned out to be much more [of] a malignant diagnosis. […] That's really a concern because some elective procedures actually end up as non-elective emergencies’.*

Regarding the postponement of elective surgical interventions and therapies, the concept of *selectivity* was often described as problematic, as it was mostly equated with *non-essential.* Experts from Türkiye, Oman, Canada, Singapore and the USA emphasized that the designation of a procedure as elective does not automatically exclude the existing necessity or (potential) urgency of this procedure. On the contrary: especially in the case of oncological diagnostics and therapies, delayed detection can have grave consequences.

Furthermore, numerous experts, especially from LMICs such as India, Zambia, Nigeria, Ecuador and Mexico, elucidated the negative effects on essential diagnostic examinations in the field of infectious diseases, such as HIV and TB:

*‘We are losing more people from HIV, from TB, from malaria [than from] COVID. But COVID, it has created this huge public health, global public health emergency and all the attention is on it […] Now we have to reallocate some of the wards … from HIV into COVID. So, but what will happen then to these programs of HIV and TB?’ (Zambia)*

As the statement shows, international standards were often used as a guide in a global crisis. This, in a number of cases, led to a lack of contextualizing national situations and needs of healthcare systems of respective countries.

Finally, the analysis of interviews with interview partners from South Africa, India, Argentina and Nigeria indicated that the pandemic might also undermine years of public work and education, for example, in the fields of vaccination or reproductive medicine. For example, the number of registered abortions and of pregnant women seeking care in hospitals reportedly dropped significantly during the first and second waves of the pandemic:

*‘In the southern part of the world where [there are] campaigns of vaccination and campaigns for the children, and campaigns for letting the young women to go and to check their pregnancies, we have achieved so much in sexual and reproductive health during the last ten to fifteen years. It was all lost […] because of COVID.’ (Argentina)*

### Health literacy

The high risk of population infection in a pandemic creates ethical conflicts for PH policy. In the context of health-influencing factors such as living space, education, nutrition and social environment, various experts mentioned the decisive influence of health literacy. Regarding the implementation of pandemic protection measures, the ability of the public to comprehend medical information seemed essential. Experts from LMICs such as Ecuador, Nigeria and India noted a health literacy gap between rich and poor. In countries without a functioning healthcare system, health literacy in the predominantly poor population was considered very low. This was not only seen due to the lack of education, but also due to dissatisfaction with the government and the daily struggle for survival, regardless of the SARS-CoV2-virus:

*‘Sixty percent of the population has no access to money,...to health [or] education. I would say [...] the culture of health is not good in my country […]. Some people from the media to the rich [classes] can understand, can be aware of taking care.’ (Mexico)*

Our results showed that also in HICs, including the United States, Canada, Poland, Sweden and Israel, health literacy was seen as more deficient in marginalized and often financially weaker groups, such as migrants and ethnic minorities. Thus, education and social background has been identified as relevant factor in the context of health literacy:

*‘It's not exactly shocking that someone who grows up in an environment like that is not getting access to the same resources than someone who's living in a […] wealthy neighbourhood.’ (USA)*

Experts from many countries, including the UK, Australia, Sweden, Ecuador and Paraguay, explained that being linguistically, socially and culturally separated from the majority society led to the lack of access to relevant information for minorities in general. In addition, some interview partners emphasized the key role of religious rites and gatherings, which showed a diminishing effect on the establishment of health competence and thus on the implementation of necessary pandemic protection measures. For example, experts from Singapore, Israel, South Korea, but also from Colombia and India stated that the realization of rituals and customs often outweighed compliance with restrictions in their importance, also pointing to a specific influence of cultural attitudes regarding health literacy.

Especially Latin American interview partners (from Ecuador, Paraguay, Mexico and Argentina) ranked interpersonal relationships and physical proximity as a significant influence on the acceptance of protective measures. This indicated a cultural influence on the representation of conflicts:

*‘The masks and the isolation of the patient in the hospital […] is also something that for our culture is terribly heavy […] so we are anything but puritan, we like human contact and for us that thing has been a very important issue’. (Argentina)*

### Inequality and vulnerable groups

The COVID-19 pandemic disproportionately affected vulnerable groups, both highlighting and exacerbating existing social and economic inequalities. This section presents experts’ perspectives on ethical challenges in LMICs stemming from high poverty. Below, we look at the rationale for and consequences of public safety measures with a focus on the vulnerability of women and children affected by lockdowns. 

#### Poverty as a specific challenge for LMICs

Higher poverty rates in LMICs were identified as the root of many difficulties. To minimize infections, countries mandated protective measures including masks and social distancing mandates as well as restrictions on mobility. Numerous experts, primarily from Latin American and African LMICs such as Ecuador, Mexico, Zambia and Tunisia, noted that lockdowns in particular created existential problems for the poor because, for example, street vending is the only source of income for many. Because many families' food choices often depend on their daily earnings, some experts talked of *‘hunger vs. COVID’.* An expert from Argentina reported in that context:

*‘Because of the high poverty levels – we live from hand to mouth, people have to wake up every day and they get daily wages from selling, [...] so you eat what you can make in one day and, the next day you die. So now the ethical issue is … can we have even say a one week shutdown? [...] the consequences would actually be worse’.*

Moreover, experts from India, Zambia, Argentina and Colombia, among others, concurred that informal employment relationships presented difficulties in the context of any lockdown. Those employees working in, for example, informal street vending were not only restricted in selling goods, but also lost customers. Given the absence of the laws protecting employees, numerous experts from LMICs reported an increase of unemployment, resulting in a sharp rise of poverty and hunger.

Furthermore, interview partners from Tunisia and India stressed that the state had imposed restrictions without offering support to affected citizens. This kind of reciprocity and fairness regarding pandemic measures should be indispensable, experts said:

*‘People were also starving because they had no income, because the entire economic activity had come to standstill. And the government did it without having an ethical obligation of caring for them. [… But] if you take measures [restricting] peoples' rights […] then you must reciprocate it by providing them support so that they can survive.’ (India)*

Data collection from December 2020 until March 2021 with experts from Türkiye, Brazil, Paraguay and Ethiopia showed also their concern with the lack of global solidarity regarding the distribution of COVID-19-vaccines. Since vaccine distribution only began in mid-December 2020 [35], interviews conducted in November 2020 did not address this topic.

A lack of global solidarity heightened inequalities between LMICs and HICs. Exclusively experts from underserved countries such as Nigeria, Pakistan, Paraguay and Ethiopia, among others, reported the unequal distribution and thus contextualized an expression of unfair conditions:

*‘I think for developing countries that's another ethical concern […] the vaccines are expensive [and there is some] scepticism about vaccines [but] assuming they work, and they are effective then the next question is: who can afford them? [In] poor countries like Ethiopia […] the government has other priorities, like infectious diseases’. (Ethiopia)*

#### Children and women as a specific vulnerable group in LMICs

In terms of specific vulnerabilities, the pandemic caused different effects globally. Although we identified several different vulnerable groups, in this paper we will present the results on children and women as a vulnerable group in LMICs and on elderly people in HICs. 

Interviewees from LMICs of Latin America and Africa, such as Mexico, Argentina, Paraguay, Tunisia and Ethiopia, explicitly named children as a vulnerable group during the pandemic. This was mainly related to school closures and nationwide lockdowns with their complex socio-economic consequences. Experts from Argentina, Brazil and Tunisia explained that lockdowns often contributed to increased domestic violence against children and women:

*‘Domestic violence against the wife, against the child […] many problems increased […] the lockdown is good perhaps for the epidem[ic] but it is not good for all the other aspects’. (Tunisia)*

Additionally, experts from Argentina and Paraguay noted that kids’ suddenly no longer having access to a free lunch in educational institutions as another negative consequence of school closures. Especially poorer families in LMICs cannot ensure sufficient nutrition for the children, thus lockdowns entailed grave consequences for health. Experts agreed that the pandemic also took away school as a safe daycare option and as a component of a stable daily routine and social environment. Moreover, as children could not play much with each other, nor interact with new children, their socio-emotional development was severely impaired. Consequently, socio-emotional skills may have developed less well, according to experts from South Korea and Ethiopia.

Women were identified as another vulnerable group by several interview partners from America, Asia, Europe and Africa. On the one hand, women’s and especially mothers’ traditional gender role –housework and the upbringing of children – meant that the pandemic increased their workload:

*‘Most of the nurses that we have, seventy percent of nurses [are women]. On one hand, we have nurses […] working extra hours, [being] most exposed to the virus and […] sometimes they are mothers that take care of [their own] children. [Also] domestic violence increases’. (Ecuador)*

Interviewees also mentioned increased domestic violence, particularly against women and children. They saw the reasons mainly in the intensification of tensions within households during the prolonged confinement of families at home, economic insecurity and lower social interaction. On the other hand, experts from LMICs such as Ecuador and Argentina stressed the very high proportion of females in care-associated professions. Hence, women had a higher workload while also bearing an increased risk of exposure both COVID-19 and domestic violence.

#### Older people as a specific vulnerable group in HICS

Experts from HICs stressed above all the vulnerability of the aging population. As shown in the following, this relates mainly to the cultural bias against the elderly in Western countries as well as to the vulnerability of residents in nursing homes, especially regarding SARS-CoV-2.

Most of the interviewees from HICs identified older people as a vulnerable group in terms of pandemic effects. Experts from Israel, the USA, Australia and Italy, among others, emphasized that an already existing but hidden low esteem for old age became more transparent and explicit. They agreed that social inequality led to general health inequality for the elderly:

*‘We had, especially when there was the first wave of COVID-19, a very sad situation with the triage, with the choice of who should have the possibility to have this emergency intervention. And there was a sort of discrimination against the older people and that is people like me’. (Italy)*

Experts from Sweden, Canada, Oman and Türkiye especially concluded that the combination of medical vulnerability and so-called ‘ageism’ resulted in structural disadvantages for older people that were ethically unacceptable in their view. Having been made from a utilitarian perspective, COVID-19-policies aimed at protecting this vulnerable group from infection and at preventing a collapse of the healthcare system. However, especially in nursing homes in many countries such as Canada, Sweden and Türkiye, this planning often led to extreme social isolation:

*‘What I mean is that discriminatory stigmatizing attitude towards the elderly, [not] all of the elderly people were [treated] like this [but even now] there is a ban for the elderly people not to go out after four o'clock. [...] We have to think of how can we help them.’ (Türkiye)*

Additionally, nursing homes and long-term care facilities as place of older people have proved to be places creating immense vulnerability for their inhabitants during the COVID-19-pandemic. According to many experts, the combination of the vulnerability of older people, the fragility of long-term care facilities as unpopular places and already deficient supply structures within the nursing profession have emerged as significant challenges in HICs in America, Oceania and Europe. In this context, a bioethicist from Austria summarized the profound structural discrimination of age on a multidimensional level as follows:

*‘In the German-speaking countries we have profound age discrimination.... That means not only the old person goes into a spiral of grievance, but being old triggers grievance and that affects [also] the relatives. And the third path is the discrimination of elder-care workers’.*

Experts from HICs such as Canada, Austria, Israel, Sweden and Australia, but also from Argentina, saw one driver for immense infection and mortality rates in nursing facilities in the need of nurses being employed in several facilities at the same time, which already was a practice before the pandemic. This unfortunate situation then led to the spread of the SARS-CoV2-virus between different facilities during the crisis. In addition, experts from Austria, France and the United Kingdom identified national inequalities in the distribution of materials such as PPE and tests between hospitals and nursing homes, accentuating the vulnerability of these facilities on the supply level:

*‘It was difficult to get enough personal protective equipment, [it] was bought by the government and primarily went to the healthcare system [...] which made it difficult for nursing homes to protect their staff and their residents adequately’. (UK)*

The management of nursing homes was often criticized. Here, interviewees found it problematic that the economic motivation of institutions was of priority instead of the protection of residents. Moreover, a few experts, including those from Canada and Austria, pointed out that hospitals and care homes increasingly tended to work against each other during the crisis, for example, by hospitals refusing to admit residents of the homes. In the end, considering all aspects mentioned, dramatic deficits in the care of nursing home residents – both, medically and emotionally – emerged. According to most experts from HICs, visiting restrictions in nursing homes, which were primarily intended for protection this group, resulted in undignified treatment.

Contrary to the conflicts outlined in HICs, various experts from Latin America and Africa stated that vulnerability of the older people did not exist to the same extent as in HICs. As a reason for that difference, they named their countries’ different demographic structures and the self-reliance of caring for family members at home:

*‘I don't see that as a major problem in Pakistan because we don't have many people living by themselves. […] Most people live with their children with families, I think there is the concept that you protect your elderly’. (Pakistan)*

Still, interview partners from Argentina, Colombia and India spoke of vulnerability of the older people in terms of medical care, primarily due to of a lack of financial resources and insufficient pensions. Where healthcare systems rely heavily on private supplementary benefits, universal, fair access cannot be guaranteed, which is leading to insufficient healthcare.

### Conflicts surrounding healthcare professionals

The analysis of the results revealed specific conflicts for health professionals, especially nurses, during the pandemic. Several levels of burden, including physical and psychological stressors, moral and legal challenges as well as societal expectations, were identified by the interviewed experts across the globe.

#### ‘Front-line’ burdens

Healthcare professionals, both in clinics and in outpatient services, faced specific professional ethics conflicts. Most experts called this a *‘*front-line*’* burden, speaking, among other factors, of an exacerbation of already existing deficits in the healthcare system generally and in nursing care specifically. Experts from France and Austria mentioned funding cuts, ongoing for years:

*‘We've been chipping away at the public health system for about twenty years now and it's really starting to show, and for the last five to ten years there's been systematic protests about the lack of pay and the stress on healthcare workers’. (France)*

In this context, the majority of interview partners emphasized that nurses in particular are generally underpaid. Experts from Poland, USA, Mexico, India, Zambia and Türkiye, among others, also agreed that this results primarily from understaffing. This does not only increase staff workload but also leads to extra hours and shifts, which are associated with a reduction in the quality of care.

Several experts, including those from the US, Canada and Spain, noted temporary policies implemented in reaction to acute staff shortages. These included, for example, appealing to retired nurses and physicians for support or activating military personnel.

About half of the interviewees emphasized that at the time of data collection there had been no official measurable or significant changes, for example, regarding working schedules or payment. Hence, numerous experts agreed that the working conditions needed radical improvements:

*‘This is actually also necessary - I believe that the caregivers are to be supported and that the COVID care has shown because they are not only to be supported on the individual level but also on the organisational and societal level’. (Austria)*

#### Psychological and moral distress

In addition to the structural deficits outlined above, the analysis of the interviews accentuates psychological and emotional burdens faced by healthcare professionals.

All interviewees agreed that psychological stress in particular was a major challenge for health professionals during the pandemic. Experts, including those from Ethiopia, Pakistan, Canada and Poland, described the fundamental moral difficulties arising from the clash between the duty to help infected patients and the danger of infecting oneself through contact with COVID-19. An expert from Japan focused on the perspective of the caregivers involved:

*‘Many of the health professionals providing care for COVID-19 patients are in an ethical dilemma: they wish to provide care but their situation is very hard so they may not be able to continue their job’.*

A bioethicist from Poland commented on this from the perspective of the general public, also considering the challenges for doctors in this context:

*‘Bioethical debates basically revolve around the degree to which medical professionals have to respond to patients' needs. What level of risk they are expected to take […]. A number of people say this is just a job like any job […]. So you should not expect that doctors to be heroes. [Others say] the duty of the doctor is much stronger’.*

Several interview partners from HICs such as France, Israel, Italy and Denmark and from LMICs like Pakistan and South Africa emphasized the possibility of these conflicts turning into moral distress. Primarily fuelled by a feeling of loss of control in everyday clinical life regarding adequate care for patients, distress was increased by precarious working conditions due to missing PPE and additionally exacerbated by social expectations. Moreover, experts from Romania, Argentina, Türkiye and Canada, among others, explicitly noted that being assigned to intensive care units or other areas outside a worker’s professional expertise without prior adequate training was a major stressor in the hospital environment:

*‘In some other cities because of the lack of the medical staff [doctors and teams of] surgeons, neurosurgeons, dermatologists were involved...and those individuals were really against it because they said...we are not really trained for this situation’. (Romania)*

Several experts also reported the specific challenges of nurses mediating between political constraints and clinical reality. During the pandemic, due to their profession, nurses were not only contact persons for patients and relatives and thus responsible for helping them address their needs, they also functioned as an executor of state decisions, as interview partners from Romania, Austria and the UK noted.

According to numerous experts, the daily, intense confrontation with patients dying in one’s care and handling grieving relatives had also become drivers for psychological overload. Experts from Japan, Spain, Sweden, Paraguay and Argentina described the treatment of dying patients during the pandemic as often undignified. In South Korea, Austria and Mexico, for example, it was not possible to say goodbye to close relatives. This resulted in a sense of injustice for relatives and patients, also creating feelings of helplessness for caregivers.

In addition to their personal sense of responsibility towards patients, healthcare professionals also felt responsible towards their colleagues and their loved ones. This blurred the boundary between the professional and the private sphere even more, as they distanced themselves from family members and friends to protect them from potential infection. Not only did this cut them off from emotional respite after work, it also caused fatigue and feelings of frustration, helplessness, victimhood and vulnerability:

*‘They [doctors and nurses] are tired […] emotionally and spiritually and physically tired because some of them cannot see their own family. In order to [protect] them […] they have decided to go into the hotel facilities paid for by donors, and they don’t go home’. (Mexico)*

Finally, interview partners from Ecuador, Israel, Paraguay and France explained that it was common for healthcare professionals and notably nurses to ultimately flee their jobs in the sense of refusing to work. Moreover, numerous protests, in which medical staff publicly pleaded for stricter PH measures, have taken place, as experts from the UK and Canada, among others, stressed:

*‘Professions are constantly throwing out they're exhausted [...]. Nurses have in fact come online and said patients are suffering and what we need is better conditions for our work’. (Canada)*

## Discussion

Our findings illustrate the complexity of bioethical issues arising from the COVID-19 pandemic. We identified various political, economic and societal problems that have existed for years but were spotlighted and amplified by the pandemic. Although these challenges are known, our material reveals significant disparities between LMICs and HICs, a critical aspect often underrepresented in the bioethical discourse. By highlighting existing gaps in the current bioethical debate, our analysis contributes to critical-constructive rethinking of a more globalised perspective for bioethics. In the following discussion, we focus on three topics of high relevance for advancing discussions on global bioethics: vulnerability, the role of organisational ethics in the clinical setting and global inequalities.

### The concept of vulnerability as a relevant issue

The analysis of our results highlights the complexity of vulnerability in the context of the pandemic and shows that the concept of vulnerability was used in a highly context-specific manner. Our results say nothing about which groups in the countries studied are genuinely vulnerable but rather reflect discourse priorities generated in the media or in the professional environment of the experts at the time of the interview. Currently, although vulnerability is an acknowledged part of the field of PH ethics [24], our interviews showed that in practice, PH ethics does not sufficiently discuss the general importance of vulnerability, its contextual complexity and all its implications. The vulnerability of specific groups creates a fundamental dilemma for PH ethics and thus deserves more attention.

### Context-sensitive vulnerability

Generally, being vulnerable means to be at an increased risk of harm with reduced capacity to protect oneself [24], entailing that any person can be vulnerable in in a specific situation [36]. The concept of vulnerability is too abstract and broad to be used without further differentiation. Regarding COVID-19, different kinds of vulnerability have been discussed globally including medical vulnerabilities like age (as in ‘the elderly’) or pre-existing conditions and socially defined vulnerabilities including those of ethnic minorities and refugees [7, 37]. We differentiate the levels of vulnerability into the risk of high infection (biomedical vulnerability) and by the degree to which pandemic protection measures disproportionately affect specific groups (social vulnerability).^6^ The negative consequences of PH interventions designed to protect the general populations or specific groups can lead to vulnerabilities in highly biomedically vulnerable groups such as older people. Additionally, these interventions may also create vulnerabilities in non-biomedically vulnerable groups, as in the example of children being indirectly harmed by school closures (see Additional file 4: Overview vulnerabilities).

### Intersectional vulnerability

The focus of discussions about vulnerability was mainly on biomedically vulnerable groups during the first wave of the pandemic [25, 38] and broadened over time. Later, social inequalities like the disproportionate impact of lockdowns on women and children in LMICs became an issue. Although those are two fundamentally different perspectives, we still speak of *vulnerability* in both contexts. Older people are not only biomedically but also socially vulnerable. When social isolation occurred due to social-distancing policies [39], social and biomedical dimensions necessarily intersected.

Our results highlight the lack of a consideration of pandemic-related vulnerability in the public bioethics discourse. As the immediate focus is either on the consideration of only medically vulnerable groups or on only socially vulnerable groups, their intersection is less often an issue. However, since biomedical and social vulnerability are directly and indirectly related, PH ethics must discuss both separately and in their intersection. Furthermore, it is necessary to consider context-specific vulnerability: every country and every region has its own kinds of vulnerable groups requiring special attention [40]. The phenomenon of vulnerability also demonstrates the transnational connections of national conflicts [37].

### Reducing vulnerabilities

The discourse cited here may still sound abstract, but Marckmann et al. (2015) stress the necessity of providing practice-oriented analyses that use a structured methodology for applying normative criteria to PH issues [41]. As PH interventions in a pandemic are meant to protect population health, they should also be designed to avoid exacerbating vulnerabilities and inequalities [41]. We showed that this was not always the case. In a pandemic that required a public and global health ethical focus, reflection of and protection of vulnerable groups should be elevated to an explicit PH ethical objective.

### Organisational ethics needed

Our results indicate the COVID-19-pandemic exacerbated already-existing precarious circumstances and the multifaceted physical and psychological challenges faced by health professionals globally. While the interview analysis reveals that all healthcare workers were under increased stress, our discussion will centre on the role of nurses. They occupied a special position in the pandemic linking the medical profession, patients and society and, in our opinion, are still underappreciated. Additionally, with the steadily growing world population and increased life expectancy worldwide, nursing care in particular is playing an increasingly important role [42, 43].

Understanding the impact of the pandemic on nursing means understanding norms of internal professional collaboration, hierarchies within health services and the distribution of professional responsibilities. All of these touch on organisational ethics, which involve behaviour-guiding moral principles like individual rights, self-interest and social responsibility and norms like honesty, fairness and compliance with legal and social obligations [44]. The ethical obligation of professional care is conventionally thought of as based on the principle of beneficence and thus recognizes a special moral obligation of nurses to advance patients’ well-being [45]. Yet the additional burdens of societal medical emergencies cannot be borne by nurses alone, especially given their precarious working conditions [46–48].

What can we conclude from these results for future bioethical debates? Overall, it is of great relevance to establish concepts of organisational ethics regarding nursing in order to reflect actual professional routines in different settings and organisations [45, 49]. The complexity of the organisation of nursing should be respected. Nursing practice is affected by a broad range of organisational differences, including for example, those between community and in-home settings or between long-term and short-term care [45].

### Essential reflection on responsibilities

Professional responsibility involves individual responsibility within the caregiver-patient relationship, but it also entails responsibilities within organizations and other collectives. The latter highlights the need for nurses to work collectively within the healthcare system to fulfil their professional obligations [50]. In essence, in nursing, ‘responsibility’ involves determining the cause of problems, assigning accountability for outcomes and distributing accountability among involved parties. It also encompasses the commitment to the whole setting, the duty to accomplish nursing tasks with empathy and awareness for moral decision-making [50]. For this to happen and to empower nurses to recognize and fulfil their responsibilities, however, a fundamental restructuring of nurses' roles must take place at the macro (health policy), meso (clinic) and micro (ward) levels.

On the macro level, the general inclusion of administrative and specific nursing ethics is needed to sharpen professionalization and actualise the assumption of professional responsibility by considering the expertise of nurses in the design of all care processes [45, 51]. Nurses’ own reflection on the ethical dimensions of their work should lead to their involvement in all significant decisions related to patient care. In addition to professionalizing the field of nursing, the development of a framework for nursing ethics would also contribute to broader discussions on equity and justice in healthcare [49, 52].

On the meso and micro levels, many of our interviewed experts concurred with several studies regarding the numerous already-existing problems that were exacerbated by the COVID-19 crisis [29, 53, 54]. The pandemic showed how conflicts arose when the overlap of responsibility was not fully defined and thus often rested with nurses, whose authority was increased by their daily patient contact and by their mediating role between patients and the doctors. A fundamental change in terms of organisational and nursing ethics would be key for defining the ethical environment [55]. This would not only benefit nurses but also contribute to their ability to act ethically, fairly and with dignity towards patients and thus to meet the goals of PH ethics.

### Global inequalities and global solidarity

Achieving equality at the level of global health is one of the greatest challenges in contemporary times. Most global health conflicts are subject to structural, cultural, social, political, historical and economic determinants within individual countries [56]. The COVID-19 pandemic has brought into focus the importance of considering national circumstances and contextual factors in formulating effective pandemic strategies [57]. Hence, global inequalities call for global solidarity as a collective response to address pressing social and economic disparities across the world.

### Increasing awareness of global inequalities in HICs

Global ethics is of enormous significance in a pandemic. The challenges national governments are likely to face, such as vaccine distribution, the protection of vulnerable groups or resource scarcity all have global dimensions [58, 59]. For instance, significant disparities exist between LMICs and HICs in terms of their access to goods traded on international markets. This leads both to shortages and surpluses of medical care resources and technologies. While various interviewed experts from HICs took issue with the postponement of elective measures, the shortages mentioned in LMICs resulted in supply-chain breakdowns for truly essential goods such as primary healthcare or even food. Hence, global inequalities in the starting conditions hindered the handling of the novel virus [57, 60]. Increasing awareness of these disparities, especially among the perspective of the more powerful HICs, is the necessary first step to pave the way towards greater equality between LMICs and HICs.

### Reliable global governance and international cooperation

Global health inequality is a well-known problem. Ruger (2009), for example, argues that global health justice requires prioritizing responsibilities through shared health governance to reduce inequalities in healthcare capacity. This points to the importance of governance structures and political power on national and international levels [61]. Precisely this international competence was lacking in the Corona pandemic, as the WHO was quickly marginalized by the more powerful states such as the USA and China. Its 2005 International Health Regulations [62] once designed to regulate such a situation, were quickly dismissed [63, 64].

Any pandemic must be understood as global crisis, but interviewees from LMICs criticized the lack of international cooperation. As we have seen during COVID-19, the global contribution of vaccines suffered from national politics, despite indications that public opinion in HICs, such as the USA and Germany, would support more utilitarian and egalitarian allocation rules [65]. Many countries prioritized their own interests, such as securing their own PPE and vaccines, rather than developing equitable distribution strategies for sharing scarce resources. This behaviour was understandable in the initial period [59], but a global crisis requires a global ethical, solidarity-based response. Active cooperation between countries is necessary to control pandemics in future scenarios [66, 67]. If only those countries with the means to stockpile supplies and buy vaccines can protect their populations, discrepancies and asymmetrical power structures between HICs and LMICs will widen [59, 68]. The COVAX-Initiative [69] for global equitable distribution of COVID-19 vaccines was a sensible idea in theory but fell short due to insufficient support. As of January 2021, 90 million doses had been administered worldwide but only 25 in sub-Saharan Africa outside of vaccination trials [67]. From a global ethical perspective, developing detailed strategies for fair allocation is necessary, but so too is a consideration of national and international governance structures including their political impact and the role of expertise in policy making.

### Limitations

A first limitation of this study is that we identified experts online and via publications; thus the definition of expertise may be biased by our language abilities and by the experts’ international visibility. To overcome this limitation, we also used a snowball-recruitment system which extended our contacts with experts less visible in those international bioethical discourses we are familiar with. Another selection bias may have been introduced because some experts declined participation due to fear of political retribution. That some interview partners requested anonymity indicates this may have been an issue.

Second, we interviewed in the rule only one expert per country. Therefore our study cannot claim quantitative representativity; rather, our focus was on taking an explorative snapshot of various experts’ opinions during one critical episode of the pandemic [70–72]. Our primary objective was to make a broadly comparative and exploratory contribution to the bioethical discourse related to the pandemic under the constraints of time and uncertainty.

Finally, we tried to overcome the European perspective on the pandemic through comparison across global regions. However, our approach may still introduce Eurocentric bias through in the study design itself. Through constant self-reflection and communication within the research group, we tried to minimize this potential bias.

## Conclusion

We conducted a qualitative study on the opinions of bioethics experts regarding bioethical issues arising from the COVID-19 pandemic in international comparison. We found that perceived ethical challenges were similar across the globe but that their perceived consequences and causes show country-specific cultural, infrastructural and economic differences. In summary, the purpose of seeking a deeper understanding of a globalised perspective on ethical issues related to pandemics is essential for crafting more effective and equitable preparedness and responses to future global health crises. It might help policy organisations and health policymakers to identify potential ethical problems and develop ethical frameworks and related strategies at early crisis stages – regarding human rights, fair allocation of scarce resources, the need for more contextualized health literacy, among others, to address them in advance. Lastly, the connections linking countries and populations during a pandemic situation were highlighted, underscoring the importance of international collaboration. Identifying specific needs can promote cooperation among nations and organisations to collectively address the bioethical challenges of pandemics. Although no single study of expert opinion can capture the full complexity of the pandemic and its global impact, our findings open a window onto the pandemic’s ethical ramifications and should motivate empirical bioethicists, in collaboration with PH experts, to continue the analysis of experts’ insights so as to be better prepared for the next pandemic.

### Supplementary Information

**Additional file 1.** Interview Guideline.

**Additional file 2. ** Code overview.

**Additional file 3. ** Summary results.

**Additional file 4. **Overview vulnerabilities.

## Data Availability

The datasets used and/or analysed during the current study are not publicly available because they could reveal the identity of interview partners. Data is available from the corresponding author upon reasonable request.

## Abbreviations

COVID-19: Coronavirus disease 2019
HIC: High-income country
HIV: Human immunodeficiency virus
LMIC: Low and Middle-income country
PH: Public health
PPE: Personal protective equipment
TB: Tuberculosis
SARS-CoV-2: Severe acute respiratory syndrome coronavirus type 2
UK: United Kingdom
USA: United States of America

## Footnotes

1 The nursing profession diverges internationally. By ‘health-care professionals’ we mean all staff working in health institutions (doctors, nurses, etc.), whereas when specifically using the term ‘nurses’ we mean trained nurses in outpatient and clinical contexts.

2 *Medicine and ethics go viral*, https://www.ethicsgoviral.com/ (01/06/2023). The project also produced a physical exhibition in the *Forum Wissen* in Göttingen from June to October 2022. For the virtual exhibition additional and new podcasts were produced based on preliminary data analysis.

3 https://data.worldbank.org/country/XO (01/06/2023). For clarity, we have combined the groups of low income, low-middle income and upper middle-income countries into LMICs. LMICs: Argentina, Brazil, Colombia, Ecuador, Ethiopia, India, Kyrgyzstan, Mexico, Nigeria, Pakistan, Paraguay, Tunisia, Türkiye, Zambia, South Africa. HICs: Australia, Austria, Canada, France, Germany, Italy, Israel, Japan, Oman, Poland, Russia, Romania, Singapore, South Korea, Spain, Sweden, United-Kingdom (UK), United States of America (USA).

4 https://zoom.us/ (01/06/2023).

5 https://atlasti.com/ (01/06/2023).

6 Also including cultural influences.

## References

[1] World Health Organization (2020). Archived: WHO Timeline - COVID-19.

[2] Dessie ZG, Zewotir T (2021). Mortality-related risk factors of COVID-19: a systematic review and meta-analysis of 42 studies and 423,117 patients. BMC Infect Dis.

[3] Killerby ME, Link-Gelles R, Haight SC, Schrodt CA, England L, Gomes DJ (2020). Characteristics associated with hospitalization among patients with COVID-19 - Metropolitan Atlanta, Georgia, March-April 2020. MMWR Morb Mortal Wkly Rep.

[4] Brauner JM, Mindermann S, Sharma M, Johnston D, Salvatier J, Gavenčiak T, et al. Inferring the effectiveness of government interventions against COVID-19. Science. 2020:1–16. 10.1126/science.abd9338. 10.1126/science.abd9338 PMC7877495 33323424

[5] Gostin LO, Friedman EA, Wetter SA (2020). Responding to Covid-19: How to navigate a Public Health Emergency Legally and Ethically. Hastings Center Report.

[6] McDaniel L. What Is Bioethics? 25.01.2021. https://bioethics.msu.edu/what-is-bioethics. Accessed 15 Oct 2023.

[7] Mastroianni AC, Kahn JP, Kass NE, editors. The Oxford Handbook of Public Health Ethics: 68 Pandemic Disease, Public Health, and Ethics. Oxford: Oxford University Press; 2019.

[8] The World Bank. World Bank Country and Lending Groups – World Bank Data Help Desk. 2022. https://datahelpdesk.worldbank.org/knowledgebase/articles/906519-world-bank-country-and-lending-groups. Accessed 14 Dec 2022.

[9] Brown RCH, Savulescu J, Williams B, Wilkinson D. Passport to freedom? Immunity passports for COVID-19. J Med Ethics. 2020:1–8. 10.1136/medethics-2020-106365. 10.1136/medethics-2020-106365 PMC7525773 32817362

[10] Wilkinson D (2020). ICU triage in an impending crisis: uncertainty, pre-emption and preparation. J Med Ethics.

[11] Reid L (2020). Triage of critical care resources in COVID-19: a stronger role for justice. J Med Ethics.

[12] Robert R, Kentish-Barnes N, Boyer A, Laurent A, Azoulay E, Reignier J (2020). Ethical dilemmas due to the Covid-19 pandemic. Ann Intensive Care.

[13] Rossouw TM, Boswell MT, Nienaber AG, Moodley K (2020). Comorbidity in context: part 1. Medical considerations around HIV and tuberculosis during the COVID-19 pandemic in South Africa. S Afr Med J.

[14] Emanuel EJ, Persad G, Upshur R, Thome B, Parker M, Glickman A (2020). Fair allocation of scarce medical resources in the time of Covid-19. N Engl J Med.

[15] Flemming S, Hankir M, Ernestus R-I, Seyfried F, Germer C-T, Meybohm P (2020). Surgery in times of COVID-19-recommendations for hospital and patient management. Langenbecks Arch Surg.

[16] Mobula LM, Samaha H, Yao M, Gueye AS, Diallo B, Umutoni C (2020). Recommendations for the COVID-19 Response at the National Level Based on lessons learned from the ebola virus disease outbreak in the Democratic Republic of the Congo. Am J Trop Med Hyg.

[17] Dunham AM, Rieder TN, Humbyrd CJ (2020). A bioethical perspective for navigating moral dilemmas amidst the COVID-19 pandemic. J Am Acad Orthop Surg.

[18] Dyer O (2020). Covid-19: Trump stokes protests against social distancing measures. BMJ.

[19] Parker MJ, Fraser C, Abeler-Dörner L, Bonsall D (2020). Ethics of instantaneous contact tracing using mobile phone apps in the control of the COVID-19 pandemic. J Med Ethics.

[20] Callaway E. The unequal scramble for coronavirus vaccines: Wealthy countries have already pre-ordered more than two billion doses. Nature. 27.8.2020;584:506–7. 10.1038/d41586-020-02450-x. 10.1038/d41586-020-02450-x 32839593

[21] Subbaraman N (2020). Who gets a Covid Vaccine first? Acces plans are taking shape: Advisory groups around the world release guidance to prioritize health-care and front-line workers. Nature..

[22] Sentell T, Vamos S, Okan O (2020). Interdisciplinary Perspectives on Health Literacy Research Around the World: More Important Than Ever in a Time of COVID-19. Int J Environ Res Public Health.

[23] Zarocostas J (2020). How to fight an infodemic. Lancet.

[24] Chadwick RF (2012). Encyclopedia of applied ethics.

[25] Carrieri D, Peccatori FA, Boniolo G (2020). COVID-19: a plea to protect the older population. Int J Equity Health.

[26] Moerenhout T. The problem in nursing homes is not Covid-19 – it is nursing homes | Journal of Medical Ethics blog. 2020. https://blogs.bmj.com/medical-ethics/2020/09/11/the-problem-in-nursing-homes-is-not-covid-19-it-is-nursing-homes/. Accessed 17 Oct 2023.

[27] Lara AM de, Medina Arellano, Maria de Jesus. The COVID-19 Pandemic and Ethics in Mexico Through a Gender Lens. 2020. https://www-1ncbi-1nlm-1nih-1gov-10nwuhna31b70.han.sub.uni-goettingen.de/pmc/articles/PMC7445801/. Accessed 8 Sep 2020. 10.1007/s11673-020-10029-4 PMC7445801 32840852

[28] Russ MJ, Sisti D, Wilner PJ (2020). When patients refuse COVID-19 testing, quarantine, and social distancing in inpatient psychiatry: clinical and ethical challenges. J Med Ethics.

[29] Iserson KV (2020). Healthcare ethics during a pandemic. West J Emerg Med.

[30] Rosenbaum L (2020). Facing Covid-19 in Italy - Ethics, logistics, and therapeutics on the epidemic's front line. N Engl J Med.

[31] Jia Y, Chen O, Xiao Z, Xiao J, Bian J, Jia H. Nurses' ethical challenges caring for people with COVID-19: A qualitative study. Nurs Ethics. 2020:1–13. 10.1177/0969733020944453. 10.1177/0969733020944453 PMC7653013 32856534

[32] McDougall RJ, Gillam L, Ko D, Holmes I, Delany C. Balancing health worker well-being and duty to care: an ethical approach to staff safety in COVID-19 and beyond. J Med Ethics. 2020:1–6. 10.1136/medethics-2020-106557. 10.1136/medethics-2020-106557 32978305

[33] Morley G, Grady C, McCarthy J, Ulrich CM (2020). Covid-19: ethical challenges for nurses. Hastings Cent Rep.

[34] Bengtsson M (2016). How to plan and perform a qualitative study using content analysis. NursingPlus Open.

[35] Watson OJ, Barnsley G, Toor J, Hogan AB, Winskill P, Ghani AC (2022). Global impact of the first year of COVID-19 vaccination: a mathematical modelling study. Lancet Infect Dis.

[36] Orth HG, Schicktanz S (2017). The vulnerability of study participants in the context of transnational biomedical research: from conceptual considerations to practical implications. Dev World Bioeth.

[37] Stok FM, Bal M, Yerkes MA, de Wit JBF (2021). Social inequality and solidarity in times of COVID-19. Int J Environ Res Public Health.

[38] Gilbert S, Why I (2020). Support Age-Related Rationing of Ventilators for Covid-19 Patients.

[39] Buss LF, Prete CA, Abrahim CMM, Mendrone A, Salomon T, Almeida-Neto C de, et al. COVID-19 herd immunity in the Brazilian Amazon. Nat Aging. 2020:26–28. 10.1101/2020.09.16.20194787.

[40] Greenaway C, Hargreaves S, Barkati S, Coyle CM, Gobbi F, Veizis A, Douglas P (2020). COVID-19: Exposing and addressing health disparities among ethnic minorities and migrants. J Travel Med.

[41] Marckmann G, Schmidt H, Sofaer N, Strech D (2015). Putting public health ethics into practice: a systematic framework. Front Public Health.

[42] OECD and European Union (2020). Health at a Glance: Europe 2020.

[43] Nations U. Population | United Nations. 23.01.2023. https://www.un.org/en/global-issues/population. Accessed 23 Jan 2023.

[44] Weber LJ (1990). The business of ethics. Hospitals need to focus on managerial ethics as much as clinical ethics. Health Prog.

[45] International Council of Nurses. The ICN-code of Ethics for Nurses: Revised 2021. 2021. https://www.icn.ch/system/files/2021-10/ICN_Code-of-Ethics_EN_Web_0.pdf. Accessed 4 Apr 2023.

[46] Waterfield D, Barnason S (2022). The integration of care ethics and nursing workload: a qualitative systematic review. J Nurs Manag.

[47] Morley G, Dierckx de Casterlé B, Kynoch K, Ramis M-A, Suhonen R, Ventura C, Arries-Kleyenstuber E. Ethical challenges faced by nurses during the COVID-19 pandemic: a scoping review protocol. JBI Evid Synth. 2023;21:970–6. 10.11124/JBIES-22-00247. 10.11124/JBIES-22-00247 PMC10173941 36692443

[48] Kartsonaki MG, Georgopoulos D, Kondili E, Nieri AS, Alevizaki A, Nyktari V, Papaioannou A (2023). Prevalence and factors associated with compassion fatigue, compassion satisfaction, burnout in health professionals. Nurs Crit Care.

[49] Deutscher Ethikrat. Patient Welfare as an Ethical Standard for Hospital Care: Opinion. 2022:1–143.

[50] Wöhlke S, Ruwe G. Uncertainties and Coping Strategies among Nurses During the First Wave of Covid-19 in Germany – Nursing Students’ Use of Diary Entries to Document their Experiences during the First Wave of Infections in the Covid-19 Pandemic. 2023. https://www.enhe.eu/archive/2022/5646. Accessed 17.10.23.

[51] Torkaman M, Heydari N, Torabizadeh C (2020). Nurses' perspectives regarding the relationship between professional ethics and organizational commitment in healthcare organizations. J Med Ethics Hist Med.

[52] Kuhse H (1997). Caring: Nurses, women, and ethics.

[53] Al-Tawfiq JA, Temsah M-H (2023). Perspective on the challenges of COVID-19 facing healthcare workers. Infection.

[54] Cadge W, Lewis M, Bandini J, Shostak S, Donahue V, Trachtenberg S (2021). Intensive care unit nurses living through COVID-19: a qualitative study. J Nurs Manag.

[55] Wlody GS (2007). Nursing management and organizational ethics in the intensive care unit. Crit Care Med.

[56] Frenk J, Gómez-Dantés O, Moon S (2014). From sovereignty to solidarity: a renewed concept of global health for an era of complex interdependence. Lancet.

[57] Ahmed T, Rahman AE, Amole TG, Galadanci H, Matjila M, Soma-Pillay P (2021). The effect of COVID-19 on maternal newborn and child health (MNCH) services in Bangladesh, Nigeria and South Africa: call for a contextualised pandemic response in LMICs. Int J Equity Health.

[58] McMahon DE, Peters GA, Ivers LC, Freeman EE (2020). Global resource shortages during COVID-19: Bad news for low-income countries. PLoS Negl Trop Dis.

[59] Ho A, Dascalu I (2021). Relational solidarity and COVID-19: an ethical approach to disrupt the global health disparity pathway. Global bioethics.

[60] Polis CB, Biddlecom A, Singh S, Ushie BA, Rosman L, Saad A (2022). Impacts of COVID-19 on contraceptive and abortion services in low- and middle-income countries: a scoping review. Sex Reprod Health Matters.

[61] Ruger JP (2009). Global Health Justice. Public Health Ethics.

[62] World Health Organization (2016). International health regulations (2005).

[63] Jones L, Hameiri S (2022). Explaining the failure of global health governance during COVID-19. Int Aff.

[64] de Souza LEPF, Castro MC, Hage Carmo E, Polidoro M (2022). The global failure of facing the pandemic. Glob Health Action.

[65] Klumpp M, Monfared IG, Vollmer S (2022). Public opinion on global distribution of COVID-19 vaccines: evidence from two nationally representative surveys in Germany and the United States. Vaccine.

[66] West-Oram PGN, Buyx A (2017). Global Health Solidarity. Public Health Ethics.

[67] Chutel L, Santora M. As Virus Variants Spread, ‘No One Is Safe Until Everyone Is Safe’. 2021. https://www.nytimes.com/2021/01/31/world/africa/coronavirus-south-africa-variant.html. Accessed 17 Oct 2023.

[68] Dalgish S. Decolonising COVID-19. The Lancet Global Health. 2020:e612. 10.1016/S2214-109X(20)30134-0 PMC7185937 32353297

[69] World Health Organization. COVAX: With a fast-moving pandemic, no one is safe, unless everyone is safe. 2020. https://www.who.int/initiatives/act-accelerator/covax. Accessed 19 Sep 2021.

[70] Mieg HA, Brunner B (2001). Experteninterviews: eine Einführung und Anleitung.

[71] Skjott Linneberg M, Korsgaard S (2019). Coding qualitative data: a synthesis guiding the novice. Qual Res J.

[72] Mays N, Pope C (1995). Rigour and qualitative research. BMJ.

