# Supplementary material for: Scarce resources, public health and professional care: the COVID-19 pandemic exacerbating bioethical conflicts — findings from global qualitative expert interviews
Source: BMC Public Health. 2023 Dec 13;23:2492. doi: 10.1186/s12889-023-17249-4 (PMC10717036; doi:10.1186/s12889-023-17249-4)
Supplement: Supplementary file 1 — Additional file 1. Interview Guideline. [file 12889_2023_17249_MOESM1_ESM.pdf]

Interview Guideline

„Medicine and Ethics go viral:

The Ethics of Covid-19-health-care

A global mapping of bioethical perspectives“

1) Please first explain briefly your expertise in bioethics/medical ethics/public health ethics. What is your personal experience and involvement in the debates about Covid-19? Could you please characterize briefly the health care system in your country?

2) If you characterized the bioethical debates because of the pandemic in your country, which ones would be the three most important ones you personally see?

3) When you consider the situation of clinics and ICU units in your country, how would you describe the main bioethical conflicts there?

4) How far are scarcity of allocation and conflicts of treatment priority between COVID-19 and non-Covid patients of concern in your country?

5) Considering the situation of long-term-care/elderly-care in your country, do you see any changes due to the pandemic and how would you describe them?

6) Thinking of public debates in your country around professional care (nurses and doctors) during the last months, which bioethical conflicts were mainly discussed in public/the media?

7) Does there exist any discrepancy between what is publicly discussed and what you and other experts see as the main bioethical problems?

8) What role do ethical conflicts between solidarity and individual freedom play in the public discourse in your country and in which concrete debates does this arise?

9) What role does the topic of discrimination (age, gender, migration background, disability, ethnic, colour, class, etc.) play in current debates in your country?

10) Do you see any conflicts concerning research ethics in your country?

11) Could you name concrete examples/situations where ethicists in your country are involved in finding solutions to current challenges?

12) What would you expect out of increased international exchange among bioethicists regarding all these challenges? What would be helpful?

13) Is there anything we have not discussed yet but should be mentioned in your opinion?
