# Supplementary material for: Scarce resources, public health and professional care: the COVID-19 pandemic exacerbating bioethical conflicts — findings from global qualitative expert interviews
Source: BMC Public Health. 2023 Dec 13;23:2492. doi: 10.1186/s12889-023-17249-4 (PMC10717036; doi:10.1186/s12889-023-17249-4)
Supplement: Supplementary file 2 — Additional file 2. Code overview. [file 12889_2023_17249_MOESM2_ESM.docx]

**Appendix 2: Code overview**

| **Main codes** and sub-codes |
| --- |
| **Academic background**  Background in bioethics  Background in public health ethics |
| **Country background**  Overview health care system  Most important bioethical conflicts |
|  |
| **Scarce resources in medical-clinical context**  Material  Personnel  Consequences of scarce resources  Reasons for scarce resources |
| **Triage**  Triage guidelines  Ethical conflicts  Levels of triage |
| **Collision with other diseases**  Non-communicable  Communicable  Acute consequences  Long-term consequences  Patients’ vs. care-facilities’ view |
|  |
| **Public health**  Public health measures  General conflicts in public health ethics  Poverty and COVID-19  Challenges and opportunities for public health |
| **Individual freedom vs. solidarity**  Description of public health ethical conflicts  Characteristics – solidarity  Characteristics – individual freedom  Concrete issues resulting from tension |
| **Vaccination**  Challenges  Global context |
| **Vulnerable groups**  Socioeconomic  Gender (women)  Cultural (indigenous groups, ethnicity, LTGBTQ)  Medical-clinical (handicapped people, elderly, …)  Others (children, prisoners, …) |
| **Health literacy**  Health promotion  Aggravating circumstances  Protests |
| **Discrepancy between experts and citizens**  Hidden topics  Challenges of discrepancy |
| **Research ethics**  Pandemic-specific conflicts  General conflicts in research ethics |
|  |
| **Solution approaches concerning bioethical conflicts**  **Chances international exchange** |
| **Ethical approaches**  Role of ethics in pandemic  Concrete involvement of ethicists  Needed approaches |
|  |
| **Exhibition codes** |
| Case study |
| Podcast |
