# Supplementary material for: Scarce resources, public health and professional care: the COVID-19 pandemic exacerbating bioethical conflicts — findings from global qualitative expert interviews
Source: BMC Public Health. 2023 Dec 13;23:2492. doi: 10.1186/s12889-023-17249-4 (PMC10717036; doi:10.1186/s12889-023-17249-4)
Supplement: Supplementary file 3 — Additional file 3. Summary results. [file 12889_2023_17249_MOESM3_ESM.docx]

**Appendix 3:** **Results summary**

**Country abbreviations:**

**LMICs:** Argentina (ARG14,17^[[1]](#footnote-1)^), Brazil (BRA), Colombia (COL), Ecuador (ECU), Ethiopia (ETH), India (IND), Kyrgyzstan (KY), Mexico (MEX), Nigeria (NIG), Pakistan (PAK), Paraguay (PA), Tunisia (TU), Türkiye (TURK), Zambia (ZA), South Africa (SA)

**HICs:** Australia (AUSL30,33), Austria (AUS2,15), Canada (CA13,22), Denmark (DEN), France (FR), Germany (GER), Italy (IT), Israel (IS), Japan (JAP), Oman (OMA), Poland (PL) Romania (RO), Russia (RUS), Singapore (SING), South-Korea (SK), Spain (SP), Sweden (SW11,24), United Kingdom (UK), United States of America (USA)

| **Topic** | **Conflicts mentioned in HICs and LMICs** | **Conflicts specific to HICs^[[2]](#footnote-2)^** | **Conflicts specific to LMICs^[[3]](#footnote-3)^** |
| --- | --- | --- | --- |
| **Effects of scarcity for the clinical context**  A1) Different dimensions of resource scarcity between LMICs and HICs | Lack of material resources in clinical setting: PPE, tests, PPE, masks, etc. (SA, ZAM, ARG17, IND, BRA, PA, TU, ETH, COL, ECU, AUS2, UK, FR, USA, AUSL30,33, RU)   - Resulting in serious COVID-19 infections among the working personnel   Financial deficiency in health care system becoming apparent   - Discrepancy of resources between urban and rural areas (JAP, ROM, PAK, KY, PA) |  | Serious infections of working personal due to lack of PPE (RUS, ZA, ARG17)  Severe lack of PPE resulting in reuse of materials (ZA, ETH, TU)  Lack of basic resources (beds, oxygen) (PAK, ETH, COL, ECU, PA, KY)  Fundamental lack of health infrastructure in country missing (MEX, KY, PA, ECU, SA) |
| **Effects of scarcity for the clinical context**  A2) Prioritization of COVID-19 patients | Prioritization of COVID-19 and emergency medicine   - Redistribution of available resources to COVID-19: conversion and closure of hospitals (IT, IND, BRA, ARG14,17) - Lack of essential diagnostics and therapy in the field of internal medicine (DN, SK, FR, IND, ECU, MEX, PA, TU, PAK, ETH, COL)   Oncological patients suffering globally: delayed diagnosis of malignant tumours due to reduced screening, diagnostics and check-ups (UK, IT, CA13, MEX, RUS, ARG14,17, ZAM, TURK, NIG, OMA, ETH, SW11)  Reasons for focus on emergency and COVID-19:   - Health care regulators and/or individual facilities aiming to minimize infections to prevent collapse of care - Patients refusing to visit potential infectious institutions, such as clinics | Postponement of surgery interventions and *“non-essential”* treatments, classified as *“elective”* (CA13,22, USA, AUS2, UK, SING, OMA):  Intra-clinical redistribution of resources to COVID-19, resulting in inadequate resources for other hospitals wards (AUS2, FR, SW11) | Lack of essential diagnostics and therapy in the field of infectious diseases (HIV, tuberculosis, malaria)  Undermining years of public work and education: vaccines, reproductive medicine (ARG17, SA, IND, NIG) |
| **Ethical challenges in the public health context**  B1) Effects of low health literacy among the public | Interconnection of social environment, age, education and health literacy  Health-influencing factors:   - Living space, education, nutrition, social environment, health literacy   Culture, religion and language barriers diminishing health literacy   - Lower health literacy among indigenous groups (AUSL30,33, ECU, PA) - Religious rites and gatherings showing a reducing effect on establishment of health literacy (SING, IS, SK, COL, IND) | Less health literacy among marginalized groups (socioeconomically weaker groups, migrants, ethnic minorities) (USA, CA13,22, ROM, PL, SW11,24, IS) | Difference in health literacy between poorer and wealthier segments of population (ETH, IND, MEX, ECU, NIG, KY, ZA, BRA)   - Lack of education - Dissatisfaction with the government - Daily struggle for survival   Culturally anchored interpersonal relationships and physical proximity significant impact on health literacy (ECU, PA, MEX, ARG14) |
| **Ethical challenges in the public health context**  B2) Prevalent poverty as a specific challenge for LMICs | Conflict: Lockdown vs. poverty due to:   - Population’s dependence on daily earnings - Informal forms of employment - State not compensating for losses imposed by restrictions |  | Poorer populations having been confronted with acute financial problems when lockdowns were imposed (ECU, MEX, ZA, TU)  Many families' nutrition often depending on everyday earnings – not possible due to restrictions – leading to the trade-off of hunger vs. COVID  Informal employment relationships presenting difficulties in the context of any lockdown:  no sales/customers + no contractual security – increase of unemployment – rise of poverty and hunger (IND, ZA, ARG14,17, COL)  Missing reciprocity from state while implementing restrictions (IND, TU)  On the international level: global solidarity missing regarding distribution of COVID-19-vaccines (BRA, ETH, NIG, TURK, PA, PAK) |
| **Ethical challenges in the public health context**  B3) Children and women as a specific vulnerable group in LMICs | Lockdown causing children’s vulnerability due to removal of school being a safe environment in LMICs  Women as a vulnerable group (SA, IT, IS, MEX, AUS15, ARG17,14, TURK, BRA, SK, PA, JAP, TU, ETH, ECU) due to:   - Traditional gender roles and its workload during pandemic |  | Children explicitly named as a vulnerable group (MEX, ARG14,17, PA, TU, ETH)  School closure leading to:   - Forced family retreat: increased domestic violence against children and women (ARG14,17, TURK, BRA, TU, ETH) - Elimination of a regular lunch in educational institutions: insufficient nutrition (ARG, PA) - Impairment of socio-emotional development (SK, ETH)   Further reasons for women’s vulnerability:   - Proportion of females in care-associated professions – increased risk of exposure (ECU, ARG17) |
| **Ethical challenges in the public health context**  B4) Older people as a specific vulnerable group | Devaluation of old age becoming more evident and explicit  Nursing homes as places of vulnerability and inadequate care (medical, emotional) for their inhabitants during the COVID-19-pandemic due to:   - Vulnerability of older people - Fragility of long-term care facilities as unpopular places - Already deficient supply structures within the nursing profession | Devaluation of old age (IS, USA, AUSL30, IT, AUS2,15, UK, SP, IT, ROM, DAN, SW11,24, CA22, TURK):   - Social inequality leading to general health inequality - Medical vulnerability and so-called ‘ageism’ resulting in structural ethically unacceptable disadvantages – extreme social isolation instead of protection (CA22, OMA, SW24, TURK)   As a cause of high infection and mortality rates in nursing facilities:  Nurses being employed in several facilities (CA22, USA, UK, SP, FR, AUS15, CA22, AUSL30/33)  Lack of cooperation between hospitals and nursing homes regarding material, competence and care (CA22, AUS2, UK, SW11)  Economically run management of nursing homes (CA22, FR) | Awareness of (TU, SA, IND) but no comparative vulnerability of older people due to:   - Varying demographic structures (SA, IS, IND, ZA, NIG, ETH, ZA, OMA, PA, PAK) - Self-reliance of caring for loved ones at home (MEX, ROM, TURK, ZA, IND, IS, NIG, OMA, TU, PAK, ECU)   Vulnerability of older people in LMICs rather due to unfair health care structures and socioeconomic inequality rather than to ageism (ARG14, COL, IND) |
| **Effects on nursing staff and professional care**  C1) Frontline burdens of nursing staff | ‘*Front-line*’ burden due to exacerbation of already-existing deficits in the nursing service sector:   - Systemic underpayment of nurses - Understaffing with subsequent additional work, extra hours, shifts   Temporary policy solutions to overcome acute shortage of personnel (CA13, POL, USA, TURK, MEX, IND, ZA) | Reasons for exacerbations: Financial shortcuts that have been taking place for years (FR)  No official measurable or significant changes regarding remuneration or schedules (USA, CA13, SP) |  |
| **Effects on nursing staff and professional care**  C2) Psychological and moral distress for nurses | Fundamental conflict: Duty to help infected patients but possibly endanger oneself through contact with COVID-19  Conflicts leading to moral distress and fatigue, burnout, frustration, etc. increased by:   - Precarious working conditions generally (underpayment, understaffing) (PL, USA, CA13, FR, DN, DE, SK, UK, MEX, IND, COL, ZA, ECU, TU, SA, ARG17) - Missing PPE (see above) - Social expectations (USA, CA13, ROM, KY, ARG17, PAK) - Missing training and demands of assignments outside professional expertise (CA13, UK, MEX, ROM, KY, ARG17, TURK, PAK) - Mediation between political constraints and clinical reality (AUS2, ROM, UK, KY, ARG17, PAK) - Intensified confrontation with people dying, often without dignity, in one’s care (SP, SW11,24, SK, AUSL30, JAP, PA, MEX, ARG17, TU, ETH, ECU) - Lack of emotional space of retreat after work due to sense of responsibility towards colleagues and own family (AUS2, IS, AUSL30, CA22, IND, SA, MEX) - Feelings of loss of control, burnout, moral distress (AUS2, FR, DN, UK, IT, IS, CA13,22, FR, SK, PAK, EHT, SA) - Consequence: protests, refusing to work (IS, FR, ECU, PA) |  |  |

1. Numbers indicate that two different experts from one county were interviewed [↑](#footnote-ref-1)
2. In seldom cases also applicable to named LMICs [↑](#footnote-ref-2)
3. In seldom cases also applicable to named HICs [↑](#footnote-ref-3)
