# Supplementary material for: Scarce resources, public health and professional care: the COVID-19 pandemic exacerbating bioethical conflicts — findings from global qualitative expert interviews
Source: BMC Public Health. 2023 Dec 13;23:2492. doi: 10.1186/s12889-023-17249-4 (PMC10717036; doi:10.1186/s12889-023-17249-4)
Supplement: Supplementary file 4 — Additional file 4. Overview vulnerabilities. [file 12889_2023_17249_MOESM4_ESM.docx]

**Appendix 4: Overview of vulnerabilities in the pandemic context**
